# Endovascular treatment of gastroduodenal artery aneurysm: case report

**DOI:** 10.1590/1677-5449.190019

**Published:** 2019-11-13

**Authors:** André Luís Foroni Casas, Mariozinho Pacheco de Freitas Camargo, Carla Batista Moisés, Henrique Simão Trad, Edwaldo Edner Joviliano

**Affiliations:** 1 Universidade de Franca – UNIFRAN, Franca, SP, Brasil.; 2 Universidade de São Paulo – USP, Campus de Ribeirão Preto, Ribeirão Preto, SP, Brasil.

**Keywords:** aneurysm, endovascular procedures, vascular surgical procedures

## Abstract

Gastroduodenal artery aneurysm is a disease with low incidence that manifests in a nonspecific form in the majority of cases, which hinders initial diagnosis. Symptomatic cases may present with abdominal pains or hemorrhage secondary to rupture. In cases in which the aneurysm ruptures, prognosis is poor and mortality can reach 40%. Steps should therefore be taken to ensure early diagnosis and treatment. Although open surgical treatment is an option, over recent years there has been a growing trend to use endovascular techniques. This report describes a rare case of a young patient with a gastroduodenal artery who was successfully treated with endovascular techniques.

## INTRODUCTION

Visceral aneurysms have low incidence (0.01 to 0.2% in the general population,^1^ and gastroduodenal artery aneurysm is one of the least common (just 1.5% of all visceral aneurysms).^2^^,^ 3 In contrast to the majority of visceral aneurysms, gastroduodenal artery aneurysms tend to be symptomatic,^1^ which may comprise minor symptoms such as nonspecific abdominal pains, or even major symptoms, such as hemodynamic instability, melena, and hematemesis (when an aneurysm ruptures into an organ of the digestive system).^4^

The pathogenesis of gastroduodenal artery aneurysms is not fully understood. Trauma, arterial hypertension, and atherosclerosis have been identified as potential risk factors for these aneurysms. Other causes of development of gastroduodenal artery aneurysms are atherosclerosis, stenosis, or even congenital absence of the celiac trunk. The gastroduodenal and pancreaticoduodenal arteries are important routes of communication between the celiac trunk and the superior mesenteric artery. Increased blood flow through pancreaticoduodenal arteries, compensating for stenosis of the celiac trunk, may cause gastroduodenal artery aneurysms.^3^

Aneurysm rupture is linked with high mortality rates (up to 40%).^2^^,^ 3 When possible, early diagnosis of these aneurysms is the ideal way to avert unfavorable prognosis.^2^ Many different resources can be used for diagnosis, such as magnetic resonance angiography and angiotomography, but angiography is considered the gold standard examination, because it has high sensitivity and offers the possibility of treatment during the same intervention.^5^^,^ 6

The majority of guidelines recommend treatment of visceral aneurysms with a diameter of 2 cm or greater. However, gastroduodenal artery aneurysms justify intervention as soon as they are discovered,^1^ as there have been reports of rupture of small aneurysms and there doesn’t appear to be any clear relationship with diameter.^2^ Although open surgical treatment is effective, over recent years there has been an increasing tendency to employ endovascular treatment, because it is less invasive and response to treatment is good. Open surgery is reserved for cases with rupture and hemodynamic instability or cases with anatomy that is not favorable for endovascular repair.^3^

We describe the case of a young patient diagnosed with an aneurysm of the gastroduodenal artery and stenosis of the celiac trunk who was successfully treated using endovascular techniques.

## CASE DESCRIPTION

The patient was a 20-year-old male with a history of chronic gastritis and a hiatus hernia. He had presented complaining of episodes of frequent abdominal pains, sometimes debilitating, associated with nausea and vomiting, with onset around 10 days prior to hospital admission.

On physical examination he was in good general health, with good color, hydrated, acyanotic, no sign of jaundice, free from fever, with normal blood pressure and peripheral pulses present. He had diffuse abdominal pains on palpation and attenuated bowel sounds.

**Figure 1:**
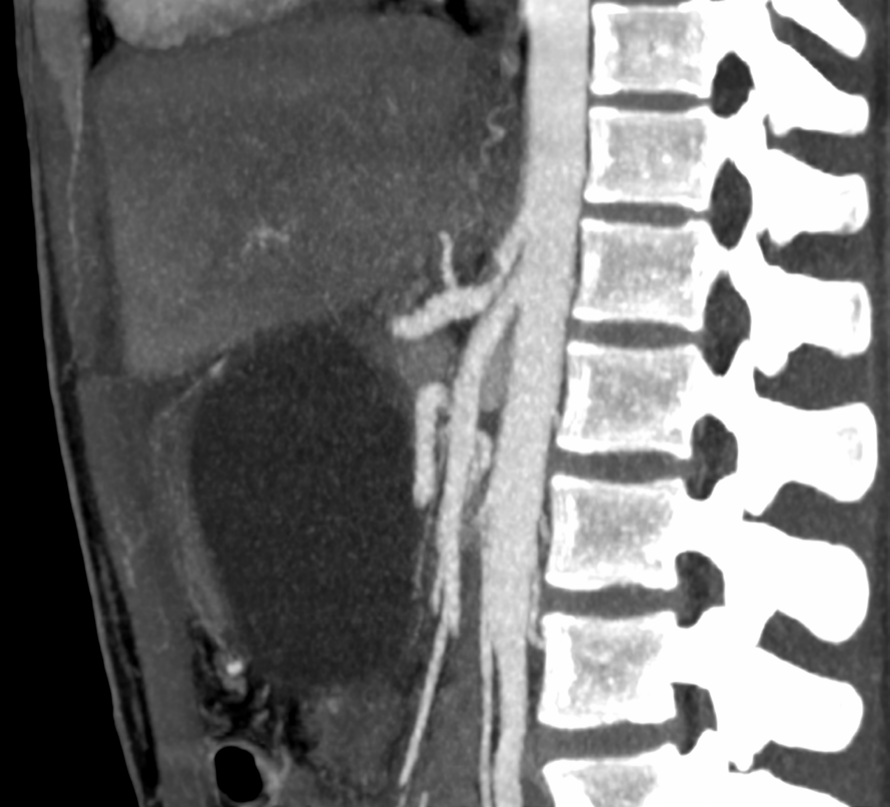
Tomography showing stenosis of the celiac trunk.

**Figure 2:**
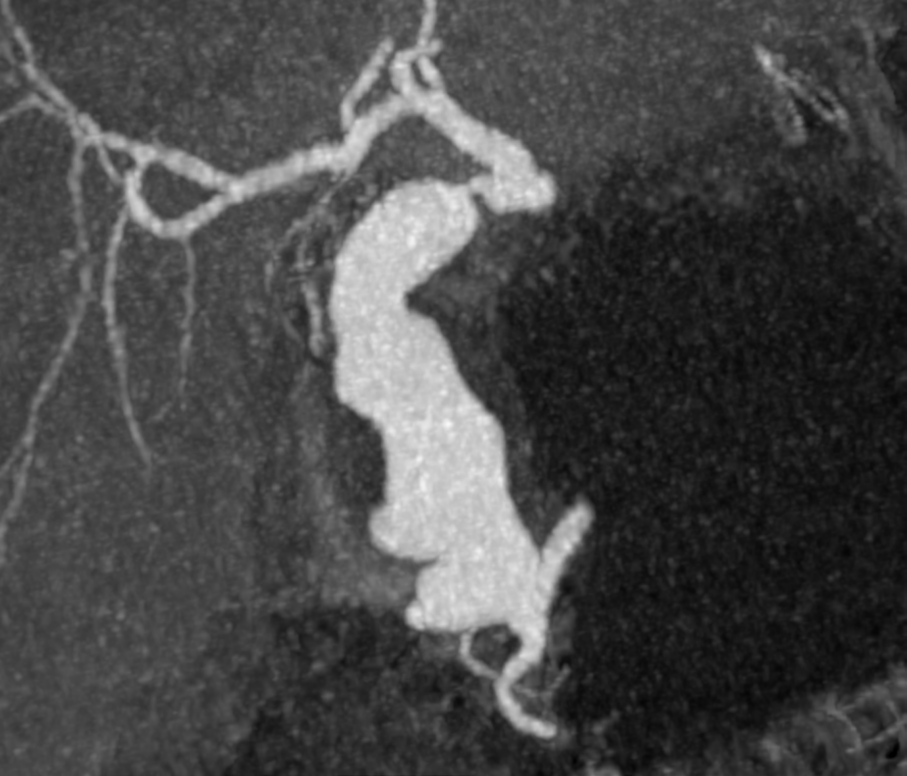
Angiotomography showing the gastroduodenal artery aneurysm.

**Figure 3:**
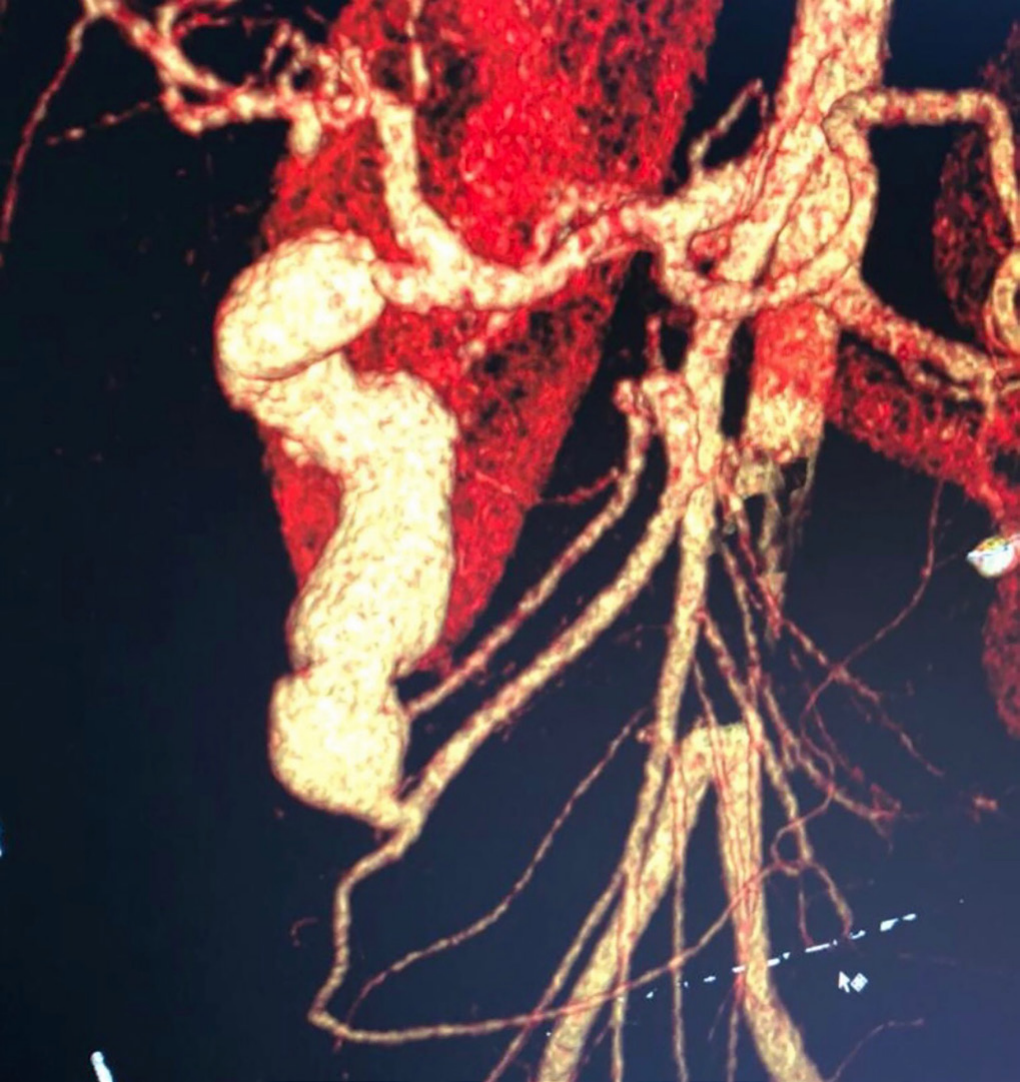
Angiotomography showing the gastroduodenal artery aneurysm.

After assessment by the general surgery team, the patient underwent computed tomography of the abdomen, which showed a gastroduodenal artery aneurysm and significant stenosis of the celiac trunk (Figure 1). The vascular surgery team was called in and angiotomography was performed, showing the gastroduodenal artery aneurysm with a maximum diameter of 3.6 cm, length of 9.5 cm and no signs of rupture (Figures 2 and 3).

**Figure 4:**
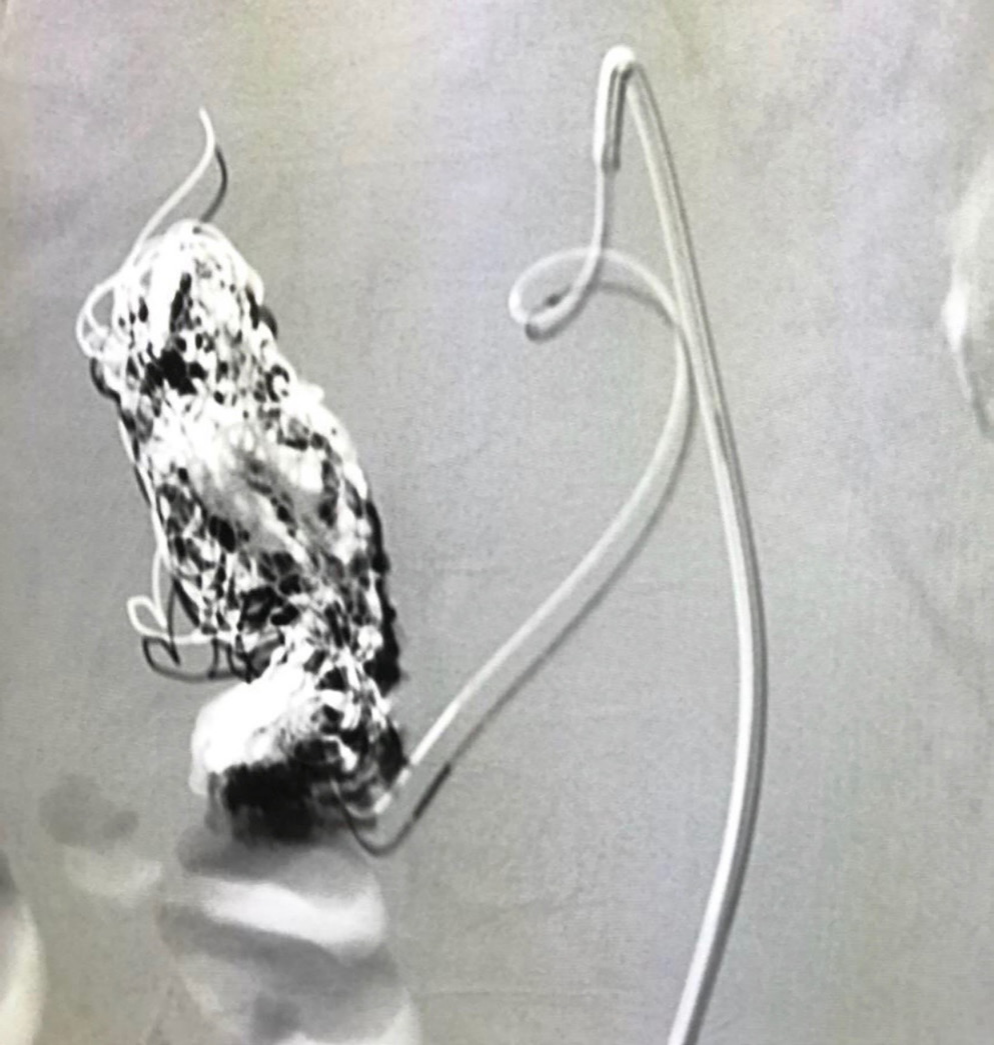
Angiography showing embolization of the gastroduodenal artery aneurysm.

**Figure 5:**
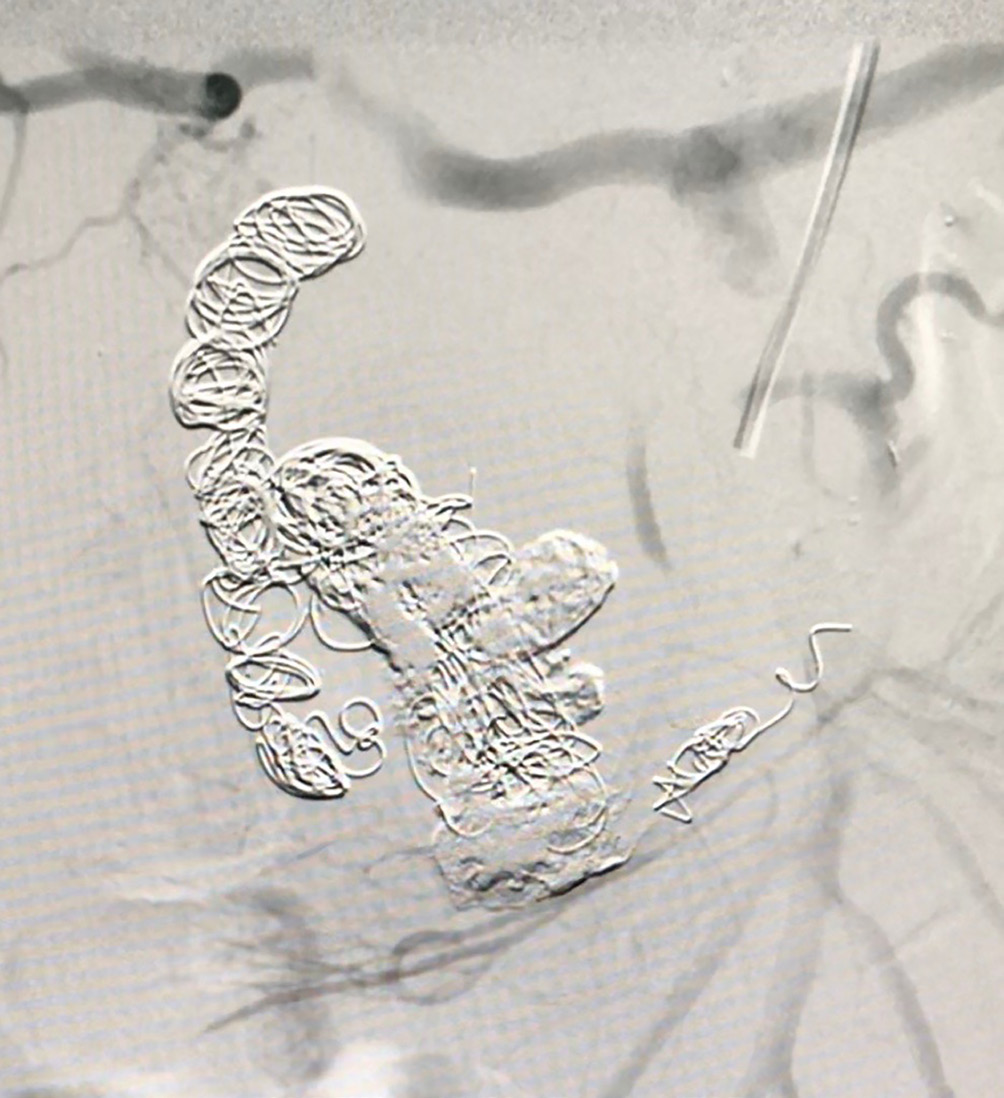
Final result of arteriography, showing exclusion of the aneurysm.

After careful consideration of the case and the patient’s general status, endovascular treatment of the aneurysm was proposed. The right common femoral artery was catheterized, a 5 French introducer was inserted and a 5 French Cobra catheter was positioned within the superior mesenteric artery (access to the aneurysm via the celiac trunk was ruled out because of the stenosis). A Rebar® microcatheter was then inserted and advanced along the path of the pancreaticoduodenal artery to access the gastroduodenal artery. Embolization was performed using a total of 19 Concerto® coils of varying sizes (6/20 mm, 8/30 mm, 9/30 mm, and 10/30 mm) and also Onyx® embolizing agent (Figure 4) and angiographic results after the procedure were satisfactory (Figure 5).

**Figure 6:**
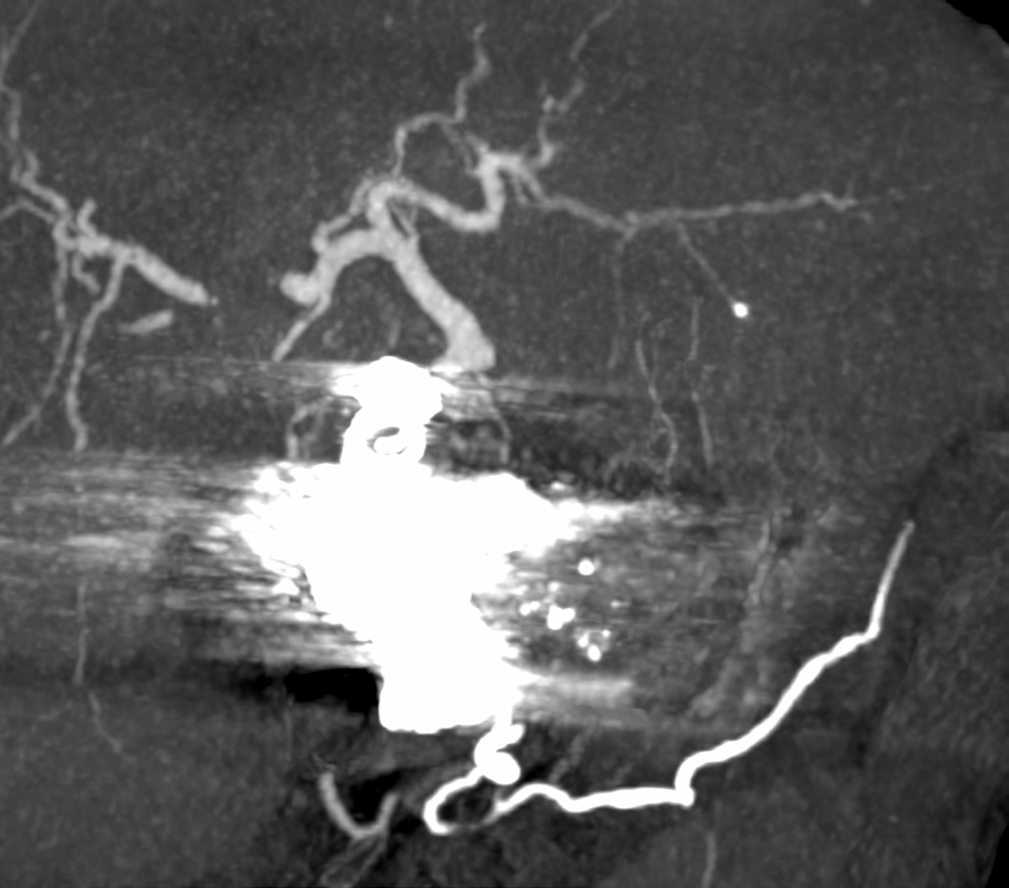
Control angiotomography.

There was significant remission of the patient’s pain after the procedure and he was discharged 2 days after surgery in a satisfactory general condition. Six months after the procedure, the patient underwent angiotomography once more, which showed complete exclusion of the aneurysm and no complications related to the procedure (Figure 6). The celiac trunk stenosis was not treated because around 12 months after the procedure the patient was still stable and asymptomatic.

## DISCUSSION

Although considered rare,^6^ visceral aneurysms are sometimes associated with fatal outcomes, primarily caused by ruptures, which have mortality rates in the range of 20 to 70%, depending on the site. Since diagnostic imaging methods are now widely available, these aneurysms are very often discovered early, while still in an asymptomatic phase (as incidental findings), in time to perform elective surgery.^7^

Stenosis of the celiac trunk, very often provoked by the arched ligament of the diaphragm (median arcuate ligament syndrome), can be the cause of formation of aneurysms, primarily because of increased retrograde collateral flow through the gastroduodenal artery and other adjacent vessels.^8^

They can be associated with acute or chronic pancreatitis, cholangitis, trauma, stenosis of the celiac trunk or iatrogenic causes.^9^ In those related to pancreatitis, activity of inflammatory mediators and proteolytic pancreatic enzymes provokes destruction of the walls of vessels in the region, leading to formation of pseudoaneurysms in the majority of cases.^3^

Alcohol abuse, prior cholecystectomy, congenital variants, Marfan Syndrome, polyarteritis nodosa, fibromuscular dysplasias, and hepatic cirrhosis have all also been described as associated factors.^5^

In contrast with the majority of visceral aneurysms, gastroduodenal artery aneurysms are generally symptomatic and can manifest with nonspecific abdominal pains (present in 46% of cases) and nausea and vomiting, or even shock, in cases in which the aneurysm ruptures.^4^^,^ 6 However, since they do not have characteristic clinical status, early diagnosis is very often difficult. Notwithstanding, in many cases, using magnetic resonance angiography and angiotomography will result in diagnosis. Angiography is considered the gold standard, not only because of its high diagnostic sensitivity, but also because it offers the opportunity to treat during the same intervention.^5^^,^ 6

When available, endovascular techniques are preferable because of their success rates (78 to 97%)^1^ and the low rates of complications and reoperation.^7^ Generally, embolization materials are employed (coils). Open surgery is reserved for cases of rupture with hemodynamic instability or cases with anatomy that is unfavorable for endovascular repair.^3^

The need to treat stenosis of the celiac trunk, when present, is still uncertain. Revascularization in this territory is performed with the intention of preventing gastrointestinal ischemia and emergence of additional aneurysms in adjacent vessels. However, because these complications have low incidence, repair of the gastroduodenal artery aneurysm only is considered sufficient.^10^
