# Supplementary material for: Endovascular treatment of gastroduodenal artery aneurysm: case report
Source: J Vasc Bras. 2019 Nov 13;18:e20190019. doi: 10.1590/1677-5449.190019 (PMC6869645; doi:10.1590/1677-5449.190019)
Supplement: Translation

# Translation: Aneurisma de artéria gastroduodenal submetido a tratamento endovascular: relato de caso

**Authors:** André Luís Foroni Casas, Mariozinho Pacheco de Freitas Camargo, Carla Batista Moisés, Henrique Simão Trad, Edwaldo Edner Joviliano

**Affiliations:** 1 Universidade de Franca – UNIFRAN, Franca, SP, Brasil.; 2 Universidade de São Paulo – USP, Campus de Ribeirão Preto, Ribeirão Preto, SP, Brasil.

**Keywords:** aneurisma, procedimentos endovasculares, procedimentos cirúrgicos vasculares

## Abstract

O aneurisma de artéria gastroduodenal é uma doença de baixa incidência que se manifesta de forma inespecífica na maioria das vezes, o que dificulta o diagnóstico inicial. Nos casos sintomáticos, pode ocorrer dor abdominal, além de hemorragia secundária à sua rotura. Nos casos de rotura aneurismática, o prognóstico é desfavorável, atingindo 40% de mortalidade. Assim, devem-se empregar os recursos necessários para o diagnóstico e intervenção terapêutica precoce. Embora o tratamento cirúrgico aberto seja uma opção, nos últimos anos, tem havido uma maior tendência ao tratamento endovascular. Relata-se um caso raro de paciente jovem portador de aneurisma de artéria gastroduodenal tratado com sucesso por meio da terapia endovascular.

## INTRODUÇÃO

Os aneurismas viscerais são doenças com baixa incidência (0,01 a 0,2% na população geral^1^, sendo o aneurisma de artéria gastroduodenal um dos menos comuns desse território (apenas 1,5% de todos os aneurismas viscerais)^2^^,^ 3. Diferentemente da maioria dos aneurismas viscerais, os aneurismas da artéria gastroduodenal tendem a ser sintomáticos^1^, podendo haver sintomas menores, como dor abdominal inespecífica, ou até mesmo sintomas maiores, como instabilidade hemodinâmica, melena e hematêmese (quando o aneurisma rompe para o interior de um órgão do sistema digestivo)^4^.

A patogênese dos aneurismas da artéria gastroduodenal ainda não é completamente compreendida. Trauma, hipertensão arterial e aterosclerose têm sido citados como potenciais fatores de risco para esses aneurismas. Outras causas descritas para o surgimento dos aneurismas da artéria gastroduodenal são aterosclerose, estenose ou até mesmo ausência congênita do tronco celíaco. As artérias gastroduodenais e pancreatoduodenais são importantes vias de comunicação entre o tronco celíaco e a artéria mesentérica superior. O aumento do fluxo sanguíneo nas artérias pancreatoduodenais como compensação para a estenose do tronco celíaco pode causar aneurisma da artéria gastroduodenal^3^.

Altas taxas de mortalidade (até 40%) estão relacionadas à perda da integralidade (rotura) do aneurisma^2^^,^ 3. Para evitar prognósticos desfavoráveis, o ideal é o diagnóstico precoce desses aneurismas, quando possível^2^. Diversos recursos podem ser empregados para esse fim, como a angiorressonância e a angiotomografia, porém a angiografia é considerada o exame padrão-ouro, por possuir alta sensibilidade e oferecer a possibilidade de se estabelecer terapêutica concomitante^5^^,^ 6.

A maioria das diretrizes recomenda o tratamento dos aneurismas viscerais quando alcançarem dois ou mais centímetros de diâmetro. Os aneurismas de artéria gastroduodenal, entretanto, justificam intervenção assim que descobertos^1^, pois foram relatados casos de roturas em aneurismas pequenos, não parecendo haver uma nítida relação com seu diâmetro^2^. Embora o tratamento cirúrgico aberto seja efetivo, nos últimos anos, tem havido uma maior tendência ao tratamento endovascular por ser menos invasivo e apresentar boa resposta terapêutica. As modalidades cirúrgicas abertas são reservadas a casos de rotura com instabilidade hemodinâmica ou casos com anatomia desfavorável para o reparo endovascular^3^.

Apresentamos o caso de um paciente jovem, com diagnóstico de aneurisma de artéria gastroduodenal associado a estenose do tronco celíaco, que foi submetido ao tratamento endovascular com sucesso.

## DESCRIÇÃO DO CASO

Paciente do sexo masculino, 20 anos de idade, com histórico de gastrite crônica e hérnia de hiato. Apresentava história de episódios de dores abdominais em cólicas frequentes, algumas vezes incapacitantes, associadas a náuseas e vômitos, com início há cerca de 10 dias da admissão hospitalar.

Ao exame físico, apresentava-se em bom estado geral, corado, hidratado, acianótico, anictérico, afebril, com pressão arterial normal e pulsos periféricos presentes. Abdome apresentava dor difusa à palpação e ruídos hidroaéreos diminuídos.

Após avaliação da equipe da Cirurgia Geral, o paciente foi submetido à realização de uma tomografia computadorizada de abdome, na qual foi evidenciado aneurisma de artéria gastroduodenal, além de estenose significativa do tronco celíaco (Figura 1). A equipe de Cirurgia Vascular foi acionada, sendo solicitada a angiotomografia, que evidenciou um aneurisma de artéria gastroduodenal com diâmetro máximo de 3,6 cm e extensão de 9,5 cm, sem sinais de ruptura (Figuras 2 e 3).

**Figura 1:** Tomografia demonstrando estenose do tronco celíaco.

**Figura 2:** Angiotomografia evidenciando aneurisma da artéria gastroduodenal.

**Figura 3:** Angiotomografia evidenciando aneurisma da artéria gastroduodenal.

Após avaliação cuidadosa do caso e do estado geral do paciente, foi proposto o tratamento endovascular do aneurisma. Por meio de cateterismo da artéria femoral comum direita e posicionando o introdutor 5 French, um cateter Cobra 5 French foi posicionado na artéria mesentérica superior (o acesso ao aneurisma através do tronco celíaco foi dificultado pela sua estenose). O microcateter Rebar® foi então introduzido, transpondo o trajeto da artéria pancreatoduodenal e acessando a artéria gastroduodenal, sendo então realizada embolização com um total de 19 molas Concerto® com tamanhos de 6/20 mm, 8/30 mm, 9/30 mm e 10/30 mm, além de agente embolizante Onyx® (Figura 4), com resultado angiográfico satisfatório após o procedimento (Figura 5).

**Figura 4:** Angiografia demonstrando embolização do aneurisma da artéria gastroduodenal.

**Figura 5:** Resultado final da arteriografia, demonstrando exclusão do aneurisma.

Houve remissão significativa do quadro álgico após o procedimento, e o paciente recebeu alta 2 dias após a intervenção cirúrgica, em satisfatório estado geral. Seis meses após o procedimento, o paciente foi submetido a nova angiotomografia, que demonstrou exclusão total do aneurisma e ausência de complicações relacionadas ao procedimento (Figura 6). Não foi realizado o tratamento da estenose do tronco celíaco, pois, cerca de 12 meses após o procedimento, o paciente mantinha quadro estável e encontrava-se assintomático.

**Figura 6:** Angiotomografia de controle.

## DISCUSSÃO

Apesar de considerados raros^6^, aneurismas viscerais estão associados, algumas vezes, a evoluções fatais, principalmente devido a quadros de rotura, que apresentam taxas de mortalidade de até 20 a 70%, dependendo da localização. Como os métodos de diagnóstico por imagem atualmente são muito difundidos, muitas vezes, esses aneurismas são descobertos precocemente, ainda em uma fase assintomática (achados incidentais), a tempo de se realizar cirurgia eletiva^7^.

A estenose do tronco celíaco, muitas vezes provocada pelo ligamento arqueado do diafragma (síndrome do ligamento arqueado mediano), explica a formação de aneurismas, principalmente devido ao aumento do fluxo colateral retrógrado na artéria gastroduodenal e outros vasos adjacentes^8^.

Podem estar associados a pancreatite aguda ou crônica, colangite, trauma, estenose do tronco celíaco ou causas iatrogênicas^9^. Nos relacionados a pancreatite, a atividade de mediadores inflamatórios e enzimas pancreáticas proteolíticas provoca a destruição da parede dos vasos da região, levando à formação de pseudoaneurismas na maioria das vezes^3^.

Abuso de álcool, colecistectomia prévia, variações congênitas, síndrome de Marfan, poliarterite nodosa, displasias fibromusculares e cirrose hepática também são descritos como fatores associados^5^.

Em oposição à maioria dos aneurismas viscerais, o de artéria gastroduodenal geralmente é sintomático, podendo se manifestar com um quadro inespecífico de dor abdominal (presente em 46% dos casos), associada a náuseas e vômitos, ou até mesmo a choque, nos casos de rotura aneurismática^4^^,^ 6. Contudo, por não apresentar quadro clínico característico, muitas vezes, é difícil realizar o diagnóstico precoce. Apesar disso, com utilização de angiorressonância e angiotomografia, em muitos casos, pode-se chegar ao diagnóstico. A angiografia é considerada o padrão-ouro não apenas pela sua alta sensibilidade diagnóstica, mas também pela possibilidade que oferece em estabelecer terapêutica concomitante^5^^,^ 6.

As técnicas endovasculares, quando disponíveis, são preferencialmente utilizadas devido às suas taxas de sucesso (78 a 97%)^1^, com baixos índices de complicações e reabordagens^7^. Geralmente são utilizados materiais embolizantes (coils). A abordagem cirúrgica aberta fica reservada a casos de rotura com instabilidade hemodinâmica ou a casos com anatomia desfavorável para o reparo endovascular^3^.

A necessidade de realização do tratamento da estenose do tronco celíaco, quando associada, ainda permanece incerta. A revascularização desse território é feita com o intuito de prevenir a ocorrência de isquemia gastrointestinal e o aparecimento de novos aneurismas nos vasos adjacentes. Entretanto, devido à baixa incidência dessas complicações, a correção do aneurisma de artéria gastroduodenal de maneira isolada é considerada adequada^10^.
